# Targeting Deep Structures to Noninvasively Modulate Fear and Anxiety

**DOI:** 10.1016/j.bpsgos.2024.100363

**Published:** 2024-09-16

**Authors:** Odile A. van den Heuvel

**Affiliations:** Amsterdam UMC, Department of Psychiatry, Department of Anatomy & Neurosciences, Vrije Universiteit Amsterdam, Amsterdam, the Netherlands; Compulsivity Impulsivity Attention program, Amsterdam Neuroscience, Amsterdam, the Netherlands

After decades of brain imaging research in cognitive neuroscience and psychiatry, initially aimed at increasing our understanding of how alterations in brain circuit function are related to deviant cognitive, emotional, and behavioral responses underlying mental disorders, there has recently been an explosion in translating this knowledge to clinical psychiatry. The application of the various neuromodulatory techniques, both invasively targeting the deep structures (such as deep brain stimulation) and noninvasively targeting cortical areas (such as repetitive transcranial magnetic stimulation [rTMS] and transcranial electric stimulation), is increasingly entering daily clinical practice thanks to its proven added value for patients who do not respond well enough to first-line treatments with psychotherapy and/or medication. An exciting new branch to the trunk is low-intensity transcranial focused ultrasound (TFUS). Unlike high-intensity TFUS, microlesioning the targeted brain structure by heating the tissue [now more commonly used in, e.g., the treatment of essential tremor (1)], low-intensity TFUS is a nonthermal neuromodulatory technique that is capable of targeting subcortical regions through sonication using multiple ultrasound pulses to modulate its neural excitability with minimal risk of tissue damage. Low-intensity TFUS regulates biochemical processes such as inflammatory responses and neurogenesis (2). Whereas well-powered randomized controlled trials and meta-analyses of the previously mentioned neuromodulation techniques (deep brain stimulation, rTMS, transcranial electric stimulation) have shown their efficacy with well-known safety profiles, TFUS is still in its infancy, with an undecided position in the clinical application repertoire. As recently reviewed by Qin *et al.* (2), most work has been done with animals, followed by some studies with healthy control participants (focusing on motor function, pain, and cognition) and only a few studies conducted with clinical populations, such as participants with depression and chronic pain.

SEE CORRESPONDING ARTICLE NO. 100342

In a new study in *Biological Psychiatry: Global Open Science,* Hoang-Dang *et al.* (3) report on their very innovative within-participant crossover proof-of-concept study in which they applied a single session of low-intensity TFUS to the right amygdala (and the left entorhinal cortex as control target) in a relatively small and old adult sample of healthy participants. Focusing on the amygdala, their aim was to reduce anxiety, evaluating its effect using both subjective measures (e.g., self-reported valence and arousal during an emotional reactivity and regulation task) and objective measures (e.g., heart rate). In their recent previous publication based on the same sample (4), they have already shown that low-intensity TFUS of the right amygdala selectively increased perfusion in the targeted brain region and selectively modulated the targeted functional frontolimbic network connectivity. Their current results add to the unexpected direction of their findings by showing that low-intensity TFUS of the right amygdala significantly increased (instead of decreased) self-reported arousal in response to negative images and heart rate during the emotional reactivity and regulation task. It did not increase overall state anxiety.

Their results are in contrast to those of another recent study that used single-session low-intensity TFUS to modulate fear processing in healthy participants (5). Instead of the right, Chou *et al.* (5) targeted the left amygdala. Compared with the sham condition, active low-intensity TFUS of the left amygdala was associated with decreased activation during fear processing in the amygdala, hippocampus, and dorsal anterior cingulate cortex, and the decrease in amygdala activation correlated with decreased subjective anxiety (5). Active (compared with sham) TFUS also resulted in decreased amygdala-insula and amygdala-hippocampal resting-state functional connectivity and increased amygdala–ventromedial prefrontal cortex resting-state functional connectivity (5). Thus, both studies show that it is possible to modulate emotional processing in humans using a single session of low-intensity TFUS of the amygdala. However, for the clinical implications of low-intensity TFUS in psychiatric disorders, the direction of the effect is crucial. The fact that these 2 studies showed opposite effects may be explained by many factors that contribute to the neural and behavioral outcomes of neuromodulation, such as the target itself (left vs. right amygdala, or its specific subregions), the target engagement during stimulation (i.e., oscillatory state and mental state affecting the direction and size of the TFUS effect), and specific choices regarding the sonication parameters, among others.

Apart from the direction of the effect, to have real clinical impact, the TFUS-induced effect should also be durable. To achieve long-term effects in clinical populations, multiple sessions of low-intensity TFUS are probably needed. Whether repeated sessions of low-intensity TFUS targeting the same region are just “modulatory” and not “lesioning” awaits further in-depth investigation of the short- and long-term risk profile. To establish reliable clinical guidelines for safe and effective application of low-intensity TFUS in psychiatry, several other issues need to be addressed apart from the direction and durability of the effects. The investigation of dosage of acoustic waves is most important. Also, the sonication parameters that mediate the treatment effect should be clarified, such as the acoustic intensity, pulse repetition frequency, pulse duration, number of bursts, and stimulation duration (2).

Another relevant clinical question is whether it is really necessary to target deep brain structures such as the amygdala directly or whether the same modulatory effect on the involved brain circuit (including the deep brain structures) can be reached by stimulation of a superficial (i.e., cortical) hub of the relevant brain circuit, for example using rTMS. Multiple experimental rTMS studies on the mechanisms of cortical stimulation aimed at modulating neural circuit excitability in humans have shown that it is very well feasible to modulate deep structures such as the hippocampus and amygdala. Based on a placebo-controlled single-session rTMS design in healthy control participants receiving low-frequency rTMS (vs. sham) that reduced left dorsolateral prefrontal cortex excitability and patients with obsessive-compulsive disorder who received high-frequency rTMS (vs. sham) that enhanced left dorsolateral prefrontal cortex excitability, de Wit *et al.* (6) showed that cortical stimulation modulated amygdala responsiveness and frontal-amygdala connectivity during symptom provocation in patients with obsessive-compulsive disorder. Stimulating one hub in a network, whether it is deeply hidden in the brain or easily accessible at the surface, not only changes the excitability of the stimulated target region but also modulates the excitability of the whole targeted network. Therefore, both targeted modulation of the amygdala using low-intensity TFUS as in Hoang-Dang *et al.* (3) and targeted modulation of the prefrontal cortex using rTMS result in altered connectivity in the same frontolimbic circuit, with potential clinical impact for psychiatric disorders that are characterized by deviant frontolimbic circuit function, such as anxiety disorders, trauma-related disorders, and obsessive-compulsive disorder. While the efficacy and safety of rTMS in the treatment of these psychiatric conditions have already been shown, the efficacy and safety profile of low-intensity TFUS awaits further research.
